# Factors Associated with Adolescents’ Intention to Participate in School-Based HPV Prevention Campaigns in Colombia: Implications for School-Based HPV Prevention Strategies

**DOI:** 10.3390/vaccines14100870

**Published:** 2026-10-01

**Authors:** Marcela Gomez-Suarez, Maria Lorcy Monsalve-Cordoba, Lizeth-Yamile Sierra-Torres, Sandra Milena Hernandez-Zambrano, Viviam Sofia Manosalva-Castillo

**Affiliations:** 1 Research Institute, Universidad FUCS, Carrera 19 N° 8A–32, Bogotá 111411, Colombia; mgomez7@fucsalud.edu.co (M.G.-S.); mlmonsalve@fucsalud.edu.co (M.L.M.-C.); lysierra2@fucsalud.edu.co (L.-Y.S.-T.); 2 Nursing Faculty, Universidad FUCS, Carrera 19 N° 8A–32, Bogotá 111411, Colombia; smhernandez3@fucsalud.edu.co

**Keywords:** human papillomavirus, HPV vaccination, adolescents, school-based interventions, school health, health education, social media, implementation science, Latin America, RE-AIM

## Abstract

Background/Objectives: Despite the availability of safe and highly effective human papillomavirus (HPV) vaccines, vaccination coverage remains below global targets in many low- and middle-income countries. School-based HPV prevention campaigns represent a promising strategy to promote adolescent engagement in preventive health behaviors; however, little is known about the factors of adolescents’ intention to participate in these initiatives, particularly in Latin America. This study aimed to identify factors of adolescents’ intention to participate in a school-based HPV prevention campaign in Bogotá, Colombia. Methods: A cross-sectional study was conducted among 125 adolescents aged 11–17 years. Sociodemographic characteristics, HPV-related knowledge, exposure to HPV-related information on social media, and intention to receive HPV vaccination were assessed as potential factors of participation. Associations with intention to participate were estimated using Poisson regression with robust variance, and adjusted prevalence ratios (aPRs) with 95% confidence intervals (CIs) were calculated. Results: Overall, 52.8% of adolescents reported an intention to participate in a school-based HPV prevention campaign. Exposure to HPV-related information on social media showed the strongest positive association with participation (aPR = 1.80; 95% CI: 1.25–2.58), followed by accurate knowledge of the recommended age for HPV vaccination (aPR = 1.68; 95% CI: 1.10–2.57), religious practice (aPR = 1.67; 95% CI: 1.23–2.27), and intention to receive HPV vaccination (aPR = 1.49; 95% CI: 1.01–2.21). Conclusions: These findings identify factors associated with adolescents’ intention to participate in school-based HPV prevention campaigns. Integrating evidence-based digital communication with practical HPV education may inform strategies to promote adolescent engagement. Future studies should determine whether these factors predict actual participation and implementation outcomes.

## 1. Introduction

Human papillomavirus (HPV) is the most common sexually transmitted infection worldwide and the necessary cause of virtually all cases of cervical cancer [1]. It is also implicated in the development of several other malignancies, including cancers of the oropharynx, anus, vulva, vagina, and penis [2]. Despite the availability of safe and highly effective vaccines, cervical cancer remains the fourth most common cancer and the fourth leading cause of cancer-related death among women worldwide, disproportionately affecting low- and middle-income countries (LMICs) [3]. To accelerate cervical cancer elimination, the World Health Organization (WHO) has established ambitious global targets that include achieving high HPV vaccination coverage among adolescents [4,5]. However, vaccination coverage remains below these targets in many countries, including Colombia, where persistent vaccine misinformation and declining public confidence following the events in El Carmen de Bolívar in 2014 have substantially affected vaccine uptake [6,7,8,9].

Although increasing HPV vaccination coverage remains a global public health priority, vaccination alone is unlikely to achieve the WHO elimination goals without complementary strategies that strengthen adolescents’ knowledge, attitudes, and engagement in preventive behaviors. School-based HPV prevention campaigns have emerged as an effective approach to improve health literacy, counter misinformation, promote positive attitudes toward vaccination, and encourage healthy preventive behaviors among adolescents [10,11]. However, the success of these interventions depends not only on their availability but also on adolescents’ willingness to participate. Despite their growing implementation, little is known about the factors of adolescents’ intention to participate in school-based HPV prevention campaigns, particularly in Latin America. Previous studies conducted in Colombia and other countries in the region have focused primarily on HPV-related knowledge, vaccine acceptability, or parental attitudes, whereas the factors influencing adolescents’ participation in school-based prevention initiatives remain largely unexplored [12,13].

Among the factors that may influence participation, digital media deserve particular attention. Social media have become one of the main sources of health information for adolescents, creating opportunities to disseminate evidence-based information while simultaneously facilitating the spread of misinformation [14,15]. Previous studies have shown that exposure to reliable HPV-related information through social media is associated with greater HPV knowledge and stronger vaccination intentions [16]. Nevertheless, integrating evidence-based digital communication with practical HPV education represents a potential strategy that warrants evaluation in future intervention studies to assess its ability to enhance adolescents’ intention to participate and support school-based HPV prevention strategies in Colombia and other Latin American settings.

From an implementation science perspective, understanding adolescents’ willingness to participate may inform the design of school-based health interventions. RE-AIM (Reach, Effectiveness, Adoption, Implementation, and Maintenance) provides a useful conceptual framework for considering factors that may influence engagement with preventive programs [17,18,19,20]. In this study, RE-AIM is used only to contextualize the potential relevance of these findings for future implementation research. Intention to participate should not be interpreted as a direct measure of reach, which requires assessment of actual participation among the eligible target population.

Therefore, this study aimed to identify the factors of adolescents’ intention to participate in a school-based HPV prevention campaign at Colegio La Amistad in Bogotá, Colombia. We think that greater practical knowledge of HPV prevention and previous exposure to HPV-related information through social media would be associated with a higher intention to participate. By identifying factors associated with adolescents’ participation, this study provides implementation-oriented evidence to inform the design of school-based HPV prevention strategies and support adolescent-centered approaches to cervical cancer prevention in Colombia and other Latin American settings.

## 2. Materials and Methods

A cross-sectional study was conducted on multiple dates throughout the second semester of 2024 among students attending Colegio La Amistad, a public secondary school located in the Kennedy district of Bogotá, Colombia. Eligible participants were students aged 11–17 years enrolled in seventh to ninth grade who provided written informed assent together with written informed consent from their parents or legal guardians. All eligible students enrolled in the selected grades were invited to participate during the study period. Students who did not provide the required consent or who had communication or cognitive difficulties that prevented completion of the questionnaire were excluded. The school population comprised 412 students aged 9–17 years. The final analytical sample consisted of 125 adolescents aged 11–17 years enrolled in seventh to ninth grade who provided written parental or legal guardian consent and adolescent assent and completed the questionnaire. The exact number of students meeting the age- and grade-based eligibility criteria who did not participate because parental consent was not obtained was not formally recorded; therefore, a formal response rate could not be calculated.

Data were collected using a structured, interviewer-administered questionnaire designed to assess sociodemographic characteristics, HPV-related knowledge, exposure to HPV-related information on social media, and behavioral intentions related to HPV prevention. The data collection instrument was adapted from the questionnaire developed by Pat and Servín [10], which was designed and evaluated to assess HPV knowledge levels and prevention aspects in the Mexican population.

Participation in the study was voluntary. Data collection was conducted during regular school hours across both morning and afternoon shifts on multiple dates throughout the second semester of 2024.

Specific time slots were requested within the school schedule to conduct single, 30-min sessions for each participating grade level. Prior to administering the questionnaire, an introductory presentation was held with the students to explain the scope of the project and outline the activities involved in the school-based HPV prevention campaign, which was tailored directly to the student population.

The primary outcome was adolescents’ intention to participate in a school-based HPV prevention campaign. To ensure clarity, the questionnaire explicitly defined this campaign to the participants as a comprehensive school health initiative consisting of interactive educational workshops on HPV transmission and prevention, spaces to resolve doubts and counter misinformation, and activities promoting the uptake of the HPV vaccine in coordination with local health services. Responses were dichotomized as Yes (*n* = 66; 52.8%) and No/Do not know (*n* = 59; 47.2%).

Independent variables included sociodemographic characteristics (sex, school grade, school shift, and religious practice); HPV-related knowledge (knowledge of HPV infection, transmission, HPV-associated diseases, vaccination site, recommended age for vaccination, and recommended number of vaccine doses); exposure to HPV-related information on social media; internet use patterns; and willingness to receive HPV vaccination.

During the second semester of 2024, the Colombian National Immunization Program recommended a single-dose HPV vaccination schedule for immunocompetent girls aged 9–17 years and boys aged 9–14 years. From 1 October 2024, eligibility for boys was expanded to 9–17 years, while a two-dose schedule administered 6 months apart was recommended for immunosuppressed adolescents. Therefore, responses regarding the number of HPV vaccine doses were analyzed according to the categories reported by participants rather than being uniformly classified as correct or incorrect, given that the applicable recommendation depended on age, sex, immunological status, and the timing of data collection.

Willingness to receive HPV vaccination was assessed using the question “Would you get vaccinated against HPV?” This item assessed willingness toward vaccination and was not intended to determine participants’ previous HPV vaccination status.

Categorical variables were summarized as frequencies and percentages, whereas continuous variables were described using measures of central tendency and dispersion, as appropriate.

Bivariate analyses were performed to examine the association between each independent variable and adolescents’ intention to participate in a school-based HPV prevention campaign. Crude prevalence ratios (PRs) and corresponding 95% confidence intervals (95% CIs) were estimated for individual category contrasts. For variables with more than two categories, indicator (dummy) variables were generated, and the overall association of the variable with the outcome was assessed using a likelihood ratio test (LRT), from which the global *p*-value was obtained. Category-specific estimates and their 95% CIs should therefore be interpreted as individual contrasts, whereas the global *p*-value reflects the joint contribution of all categories of the variable. Variables with *p*-values < 0.20 in the bivariate analysis were considered eligible for inclusion in the multivariable model; for variables with more than two categories, this criterion was based on the global *p*-value.

A multivariable Poisson regression model with robust variance was fitted to estimate adjusted prevalence ratios (aPRs) and 95% confidence intervals (95% CIs). Because the prevalence of the outcome exceeded 10%, Poisson regression with robust variance was preferred over logistic regression to directly estimate prevalence ratios and avoid overestimation of effect sizes associated with odds ratios in cross-sectional studies [21,22].

Model fit for the multivariable Poisson regression was evaluated using goodness-of-fit measures, including the model deviance and Akaike Information Criterion (AIC), in addition to assessing multicollinearity via variance inflation factors (VIF).

Statistical significance was established at *p* < 0.05. All statistical analyses were performed using jamovi version 2.6.44 (The jamovi Project, Sydney, Australia). Exploratory interaction terms between sex and selected factors, including HPV-related knowledge and exposure to HPV-related information on social media, were evaluated in separate models.

The study was conducted in accordance with the ethical principles of the Declaration of Helsinki and Colombian regulations governing research involving human participants (Resolution 8430 of 1993 of the Ministry of Health and Social Protection). Written informed consent was obtained from parents or legal guardians, and written informed assent was obtained from all participating adolescents prior to enrollment.

The study protocol was approved by the Human Subjects Research Ethics Committee of Hospital de San José (CEISH) (Approval No. CEISH 196-2024; 7 March 2024).

This study was reported in accordance with the Strengthening the Reporting of Observational Studies in Epidemiology (STROBE) Statement.

## 3. Results

The study included 125 adolescents aged 11–17 years who completed the survey. Overall, 52.8% (*n* = 66) reported an intention to participate in a school-based HPV prevention campaign, whereas 47.2% (*n* = 59) reported that they would not participate or were uncertain. The distribution of participant characteristics according to participation intention is presented in Table 1.

HPV-related knowledge varied across the evaluated domains. Correct knowledge of the recommended age for HPV vaccination was more frequent among adolescents who intended to participate (37.9% vs. 20.3%), whereas knowledge of HPV-associated diseases was low in both groups (15.2% vs. 20.3%). Most participants were unable to identify the recommended number of HPV vaccine doses, regardless of their intention to participate.

Social media use was nearly universal among participants (>93% in both groups). However, previous exposure to HPV-related information on social media was substantially more common among adolescents who intended to participate than among those who did not (33.3% vs. 13.6%). Similarly, intention to receive HPV vaccination was more frequent among adolescents willing to participate in the prevention campaign (72.7% vs. 47.5%).

In the bivariate analysis, school shift, religious practice, knowledge of HPV-associated diseases, knowledge of the recommended age for HPV vaccination, knowledge of the recommended number of vaccine doses, previous exposure to HPV-related information on social media, and intention to receive HPV vaccination met the predefined criterion for inclusion in the multivariable model (*p* < 0.20). No statistically significant associations were observed for sex, school grade, knowledge of HPV transmission, knowledge of vaccination sites, or internet use patterns (Table 2).

In the multivariable analysis, several factors remained independently associated with adolescents’ intention to participate in a school-based HPV prevention campaign (Table 3). Exposure to HPV-related information on social media showed the strongest positive association with participation (aPR = 1.80; 95% CI: 1.25–2.58), followed by knowledge of the recommended age for HPV vaccination (aPR = 1.68; 95% CI: 1.10–2.57), religious practice (aPR = 1.67; 95% CI: 1.23–2.27), and intention to receive HPV vaccination (aPR = 1.49; 95% CI: 1.01–2.21). Conversely, knowledge of HPV-associated diseases (aPR = 0.59; 95% CI: 0.38–0.90) and reporting one dose as the required HPV vaccination schedule (aPR = 0.43; 95% CI: 0.24–0.77) were associated with a lower intention to participate. No statistically significant association was observed for adolescents who reported two doses as the required HPV vaccination schedule (aPR = 0.76; 95% CI: 0.49–1.20; *p* = 0.239).

The overall multivariable model demonstrated satisfactory fit, as indicated by the goodness-of-fit statistics. Furthermore, no evidence of relevant multicollinearity was identified among the independent variables included in the final model (all VIF values ≤ 2.1), supporting the stability of the regression estimates.

## 4. Discussion

This study identified multiple informational, behavioral, and contextual factors associated with adolescents’ intention to participate in a school-based HPV prevention campaign in Bogotá, Colombia. Unlike studies focusing exclusively on HPV vaccine acceptance, this study examined factors associated with adolescents’ intention to engage in school-based HPV prevention initiatives and explored their potential relevance to future implementation.

The findings indicate that exposure to HPV-related information on social media, practical knowledge of HPV vaccination, religious practice, school shift, and intention to receive the HPV vaccine were independently associated with greater intention to participate. Collectively, these findings suggest that adolescents’ engagement in school-based prevention initiatives is shaped not only by knowledge but also by behavioral and contextual factors that may influence adolescents’ willingness to engage in preventive interventions.

From an implementation science perspective, these findings are particularly relevant because participation represents a prerequisite for the successful delivery of school-based prevention programs. Educational interventions cannot achieve their intended impact if adolescents do not engage with them. Therefore, identifying factors associated with participation provides valuable evidence to inform strategies aimed at maximizing the reach of HPV prevention initiatives. Adolescents’ willingness to participate may inform the design of future school-based prevention programs. However, intention to participate is not equivalent to RE-AIM Reach, which requires assessment of actual participation among eligible individuals. Accordingly, RE-AIM is used here as a conceptual framework rather than as an implementation outcome evaluated in this study.

The strongest association observed in this study was previous exposure to HPV-related information on social media. While previous exposure to HPV-related content on social media showed the strongest positive association with participation intention in our study, it is critical to recognize that exposure does not necessarily equate to exposure to accurate or evidence-based information. Digital platforms function as double-edged swords: while they offer valuable opportunities for health promotion, they also serve as major sources of HPV-related misinformation and vaccine hesitancy. Because our study assessed general exposure to HPV content rather than the specific factual accuracy or quality of the information consumed, this finding must be interpreted with caution. It highlights the urgency for health authorities not only to utilize digital channels for health promotion, but to actively deploy verified, adolescent-centered digital content that counteracts online misinformation.

This finding is consistent with growing evidence indicating that digital platforms have become one of the primary sources of health information for adolescents and can positively influence HPV-related knowledge and vaccination intentions when evidence-based information is disseminated [14,16,23]. Our findings extend this evidence by suggesting that digital communication may also promote adolescents’ engagement in preventive educational activities. The above is suggested considering that some adolescents may also have been more likely to seek this type of information due to a pre-existing interest in the topic. An outcome that has received considerably less attention than vaccine acceptance. These findings suggest that evidence-based digital communication may be considered when designing school-based HPV prevention strategies. Educational videos, peer-generated content, interactive campaigns, and collaborations with trusted digital communicators may increase adolescents’ engagement while simultaneously counteracting HPV-related misinformation [17,19,24,25].

An equally important finding was the different contribution of practical and disease-related knowledge to participation intention. Adolescents who correctly identified the recommended age for HPV vaccination were significantly more likely to express willingness to participate, whereas knowledge of HPV-associated diseases showed an inverse association. This distinction suggests that practical knowledge may be more influential than disease awareness in promoting preventive behaviors. Information that helps adolescents understand when, where, and how to access preventive services may reduce perceived barriers and increase self-efficacy, consistent with established behavioral theories such as the Health Belief Model and Social Cognitive Theory [26]. By contrast, disease-related knowledge alone may be insufficient to motivate participation if adolescents do not perceive the information as personally relevant or actionable. These findings suggest that school-based campaigns should prioritize practical, action-oriented educational messages that facilitate informed decision-making rather than focusing exclusively on disease severity.

Intention to receive the HPV vaccine was also independently associated with participation in school-based prevention campaigns, reinforcing the concept that preventive behaviors tend to cluster rather than occur in isolation [27]. Adolescents who are receptive to one preventive behavior may also be more likely to engage in complementary health promotion activities. This finding highlights the importance of designing integrated interventions that simultaneously strengthen HPV knowledge, vaccination confidence, and participation in educational initiatives instead of addressing these components separately.

School shift and religious practice emerged as contextual factors associated with participation. The association observed for afternoon school attendance should not be interpreted as a direct effect of the school schedule itself but rather as a possible indicator of broader socioeconomic and contextual characteristics that were not directly measured. Similarly, although religious practice was also positively associated with participation intention, this finding must be interpreted with substantial caution. In this study, religious practice was assessed using a simple binary measure (‘Yes’ vs. ‘No’), which lacks the precision needed to capture complex, multidimensional constructs such as spiritual beliefs, frequency of religious attendance, or specific institutional affiliations. Rather than demonstrating an independent causal effect of religiosity itself, this observed association likely functions as a proxy for broader unmeasured social, family, cultural, or community support structures that facilitate youth participation in organized health activities. Furthermore, potential residual confounding by unmeasured household socioeconomic factors or parental engagement cannot be ruled out.

The present study has several strengths. It addresses an understudied aspect of adolescents’ engagement in a Latin American setting, incorporates multiple informational, behavioral, and contextual factors into a single analytical model, and applies an appropriate statistical approach by estimating prevalence ratios using Poisson regression with robust variance. Furthermore, the absence of relevant multicollinearity supports the stability and internal consistency of the final multivariable model.

This study also has important limitations. First, the cross-sectional design precludes causal inference, and reverse causality cannot be excluded. Second, data collection was conducted on multiple dates throughout the second semester of 2024 in a single public educational institution in Bogotá with a relatively small sample size (*N* = 125), which limits the external validity of the findings. Therefore, the results may not be generalizable to adolescents in other educational settings, socioeconomic contexts, or regions of Colombia. Future multicenter studies with larger and more diverse samples are needed to confirm these findings and improve their generalizability. Third, the use of self-reported measures may have introduced recall and social desirability bias. Additionally, participants’ previous HPV vaccination status was not assessed. Therefore, responses to the question “Would you get vaccinated against HPV?” may have been interpreted differently by adolescents who had already received one or more HPV vaccine doses. Previously vaccinated participants may have responded affirmatively, reflecting prior or continued willingness to receive the vaccine, or negatively because they considered vaccination already completed or unnecessary. This ambiguity may have resulted in misclassification of vaccination intention and may have influenced the observed association between willingness to receive HPV vaccination and intention to participate in the school-based prevention campaign. Future studies should assess vaccination status separately from prospective vaccination intention.

Fourth, although multiple variables were adjusted for, residual confounding by unmeasured contextual factors cannot be excluded. Finally, the relatively modest sample size may have reduced the precision of some estimates, particularly for categories with few observations. Reverse causality is particularly relevant to the observed association between exposure to HPV-related information on social media and participation intention. Given the cross-sectional design, adolescents with greater pre-existing interest in HPV prevention or health-related topics may have been more likely both to seek, notice, or recall HPV-related content on social media and to report willingness to participate in the campaign. Therefore, this association should not be interpreted as evidence that social media exposure increases participation intention.

Furthermore, because participation required both written informed consent from parents or legal guardians and written informed assent from the adolescents, the final sample is subject to self-selection and parental-selection bias. Although the school population comprised 412 students aged 9–17 years, the exact number of age- and grade-eligible students who did not participate because parental consent was not obtained was not formally recorded. Consequently, a formal response rate could not be calculated. It is possible that adolescents who participated belonged to families with a higher baseline interest or engagement in school activities and preventive health behaviors, which could potentially overestimate the overall intention to participate in the student population.

Finally, the selection of variables included in the multivariable model was based on statistical significance criteria. Consequently, some variables of potential clinical or theoretical relevance may not have been considered. Furthermore, the model was defined based on observed data rather than prior specification; therefore, the results should be interpreted with caution.

Overall, this study identifies factors associated with adolescents’ intention to engage in school-based HPV prevention initiatives. Practical HPV education, evidence-based digital communication, and context-sensitive approaches may inform intervention design. Future studies should determine whether these factors translate into actual participation and improved implementation outcomes.

Such evidence will be essential to support progress toward the World Health Organization’s goal of eliminating cervical cancer as a public health problem.

## 5. Conclusions

This study identified informational, behavioral, and contextual factors of adolescents’ intention to participate in school-based HPV prevention campaigns in Bogotá, Colombia. Exposure to HPV-related information on social media, practical knowledge of HPV vaccination, intention to receive the HPV vaccine, and selected contextual factors were independently associated with greater participation intention, highlighting that adolescent engagement is shaped by more than knowledge alone.

These findings may inform the design of school-based HPV prevention strategies aimed at promoting adolescent engagement. Integrating practical HPV education with evidence-based digital communication warrants further evaluation.

Because this study assessed intention rather than actual participation, future studies should determine whether these factors predict participation and formal implementation outcomes, including RE-AIM Reach.

## Figures and Tables

**Table 1:** Characteristics of study participants according to intention to participate in a school-based HPV prevention campaign.

Variable	Yes (*n* = 66)		No (*n* = 59)		Total (*N* = 125)
	n	%	n	%	n
SOCIODEMOGRAPHIC VARIABLES					
**Sex**					
Male	33	55.0	27	45.0	60
Female	32	50.0	32	50.0	64
Prefer not to answer	1	100.0	0	0.0	1
**Age group**					
Early adolescence	35	48.6	37	51.4	72
Late adolescence	31	58.5	22	41.5	53
**Grade**					
Seventh	24	46.2	28	53.8	52
Eighth	10	55.6	8	44.4	18
Ninth	32	58.2	23	41.8	55
**School shift**					
Morning	35	46.1	41	53.9	76
Afternoon	31	63.3	18	36.7	49
**Religious practice**					
Yes	35	67.3	17	32.7	52
No	31	42.5	42	57.5	73
HPV KNOWLEDGE					
**Do you know what HPV infection is?**					
A disease that only affects women	10	52.6	9	47.4	19
An infection that can cause cancer	33	55.0	27	45.0	60
Do not know	23	50.0	23	50.0	46
**Do you know how HPV is transmitted?**					
Yes	30	61.2	19	38.8	49
No	36	47.4	40	52.6	76
**Do you know which diseases HPV causes?**					
Yes	10	45.5	12	54.5	22
No	56	54.4	47	45.6	103
**Do you know where to get the HPV vaccine?**					
Yes	47	55.3	38	44.7	85
No	19	47.5	21	52.5	40
**Do you know at what age the HPV vaccine is administered?**					
Yes	25	67.6	12	32.4	37
No	41	46.6	47	53.4	88
**Do you know how many doses of the HPV vaccine are required?**					
1 dose	5	45.5	6	54.5	11
2 doses	24	58.5	17	41.5	41
3 doses	0	0.0	1	100.0	1
Do not know	37	51.4	35	48.6	72
SOCIAL MEDIA EXPOSURE					
**Do you use any social media platform?**					
Yes	62	52.1	57	47.9	119
No	4	66.7	2	33.3	6
**At what time of day do you connect to the internet?**					
Morning	3	42.9	4	57.1	7
Afternoon	14	40.0	21	60.0	35
Afternoon/Evening	19	76.0	6	24.0	25
All of the above	30	51.7	28	48.3	58
**How many times per week do you connect to the internet?**					
1–2 times	9	50.0	9	50.0	18
3–5 times	11	57.9	8	42.1	19
Every day	46	52.3	42	47.7	88
**Have you seen information about HPV on social media?**					
No	29	43.9	37	56.1	66
I have not searched for it	15	51.7	14	48.3	29
Yes	22	73.3	8	26.7	30
INTENTION TO BE VACCINATED AGAINST HPV					
**Would you get vaccinated against HPV?**					
Yes	48	63.2	28	36.8	76
No	18	36.7	31	63.3	49

**Table 2:** Bivariate analysis of factors associated with adolescents’ intention to participate in a school-based HPV prevention campaign.

Variable/Category	Crude PR	95% CI Lower	95% CI Upper	*p*-Value	Significance
Sociodemographic variables					
School shift (Afternoon vs. Morning)	1.30	1.00	1.69	0.096	Marginal *
Religious practice (Yes vs. No)	1.62	1.18	2.23	0.004	**
HPV knowledge					
Knows the diseases caused by HPV (Yes vs. No)	0.74	0.52	1.04	0.089	Marginal *
Knows the age of vaccine administration (Yes vs. No)	1.51	1.12	2.04	0.007	**
Vaccine doses: 1 dose (vs. Do not know)	0.58	0.28	1.19	Global *p* = 0.011	*
Vaccine doses: 2 doses (vs. Do not know)	0.89	0.60	1.31		*
Social media exposure					
Saw HPV information on social media: Yes (vs. Did not see)	1.71	1.20	2.44	Global *p* = 0.059	Marginal *
Did not search for information (vs. Did not see)	1.07	0.70	1.64		NS *
Preventive behavior					
Intention to be vaccinated against HPV (Yes vs. No)	1.46	1.04	2.04	0.031	*

NS: not significant. * *p* < 0.20. ** *p* < 0.05. PR: prevalence ratio. 95% CI: 95% confidence interval. Wald test. For polytomous variables, the global *p*-value is reported.

**Table 3:** Multivariable Poisson regression analysis of factors associated with adolescents’ intention to participate in a school-based HPV prevention campaign.

Factor/Category	aPR	95% CI Lower	95% CI Upper	*p*-Value
School shift: Afternoon vs. Morning	1.39	1.01	1.91	0.043
Religious practice: Yes vs. No	1.67	1.23	2.27	0.001
Knows the diseases caused by HPV: Yes vs. No	0.59	0.38	0.90	0.015
Knows the age of vaccine administration: Yes vs. No	1.68	1.10	2.57	0.017
Vaccine doses: 1 dose vs. Do not know	0.43	0.24	0.77	0.005
Vaccine doses: 2 doses vs. Do not know	0.76	0.49	1.20	0.239
Vaccine doses: 3 doses vs. Do not know	NE			
Saw HPV information on social media: Yes vs. Did not see	1.80	1.25	2.58	0.002
Did not search for information vs. Did not see	1.15	0.77	1.73	0.490
Intention to be vaccinated: Yes vs. No	1.49	1.01	2.21	0.046

aPR: adjusted prevalence ratio obtained by Poisson regression with robust variance. 95% CI: 95% confidence interval. aPR = 0.00: complete absence of events in the “Yes” category of the outcome in this subgroup (n = 1); the estimate is not valid. NE: not estimable.

## Data Availability

The original contributions presented in this study are included in the article. Further inquiries can be directed to the corresponding author.
